# Hypoxia and aspirin additively increase intracellular glutamine accumulation in *PIK3CA*-mutated colorectal cancer cells

**DOI:** 10.1038/s41598-026-42753-z

**Published:** 2026-03-24

**Authors:** Nodoka Umezaki, Shogen Boku, Yoshiyuki Matsuo, Tetsushi Yamamoto, Hironaga Satake, Motoki Watanabe, Kiichi Hirota, Tomoharu Sugie, Mitsugu Sekimoto

**Affiliations:** 1 Department of Surgery, Kansai Medical University, Hirakata City, 573-1191 Osaka Japan; 2 Cancer Treatment Center, Kansai Medical University Hospital, 2-3-1 Shinmachi, Hirakata City, 573-1191 Osaka Japan; 3 Department of Human Stress Response Science, Institute of Biomedical Science, Kansai Medical University, Hirakata City, 573-1191 Osaka Japan; 4 Pathological and Biomolecule Analyses Laboratory, Faculty of Pharmacy, Kindai University, Higasiosaka City, 577-8502 Osaka Japan; 5 Department of Medical Oncology, Kochi Medical School, Kochi University, Nankoku City, 783-8505 Kochi Japan; 6 Department of Molecular-Targeting Prevention, Kyoto Prefectural University of Medicine, Kamigyo-ku, Kyoto, 602-8566 Japan

**Keywords:** aspirin, *PIK3CA*, hypoxia, glutaminolysis, colorectal cancer, Cancer, Cell biology, Drug discovery, Oncology

## Abstract

Aspirin exhibits potential as a repurposed agent for preventing and treating cancer, specifically *PIK3CA*-mutated (MT) colorectal cancer. However, the fundamental biological reasons for the particular antitumor activity of aspirin against *PIK3CA*-MT colorectal cancer are not yet fully elucidated. We have previously established that aspirin relates to glutamine metabolism in *PIK3CA*-MT colorectal cancer. In evaluating the antitumor effects of aspirin, it is essential to consider the tumor growth environment, such as hypoxia. The impact of the tumor microenvironment on aspirin efficacy remains unknown. Therefore, we aimed to investigate the mechanisms through which hypoxia influences aspirin- associated intracellular glutamine accumulation in *PIK3CA*-MT colorectal cancer cell lines. Connectivity Map analysis was used to explore the associations of aspirin with biological pathways in HT-29 (*PIK3CA*-MT) cells. RNA sequencing was performed on *PIK3CA*-wild type (WT)/mutant isogenic SW48 cell lines to assess aspirin’s effects under hypoxic conditions. Targeted metabolomics was employed to measure intracellular glutamine levels in *PIK3CA*-MT HCT116 cells following aspirin treatment and hypoxic stimulation. Bioinformatic analysis revealed associations of aspirin with amino acid metabolism and hypoxic pathways. Under hypoxic conditions, aspirin enhanced amino acid uptake in *PIK3CA*-MT cells. Targeted metabolomics showed that aspirin treatment and hypoxic stimulation additively upregulated intracellular glutamine levels in *PIK3CA*-MT cells. This increase was reversed by V-9302, an inhibitor of the glutamine transporter ASCT2, which demonstrated antitumor potential in combination with aspirin. Our findings suggest that the combination of aspirin and hypoxia significantly enhances intracellular glutamine accumulation in *PIK3CA*-MT colorectal cancer under hypoxic conditions, emphasizing glutamine metabolism as a potential therapeutic target to enhance aspirin’s efficacy.

**Supplementary Information:**

The online version contains supplementary material available at https://doi.org/10.1038/s41598-026-42753-z.

## Introduction

Non-steroidal anti-inflammatory drugs (NSAIDs) present considerable potential as repurposable drugs for preventing and treating cancer, especially colorectal cancer^1,2^. Cyclooxygenase (COX) has been identified in pharmacological research as the classical target molecule for aspirin. Recently, the COX-independent anti-tumor effects of aspirin, such as mTOR inhibition, autophagy activation, and effects on the cancer microenvironment, have attracted significant research interest^3,4^. Several randomized trials investigating aspirin’s preventive impact on cardiovascular and cerebrovascular events have found that consistent aspirin use may lower both the occurrence and death rates of colorectal cancer^5–8^. Notably, aspirin has been shown to epidemiologically reduce mortality in *PIK3CA*-mutated (MT) colorectal cancer (CRC), which accounts for 10–20% of cases. Accumulating clinical evidence strongly supports *PIK3CA* mutation as a promising predictive biomarker for aspirin efficacy^9–13^. For instance, the ALASCCA study^13^, a large randomized controlled trial, demonstrated that three years of low-dose adjuvant aspirin significantly reduced the risk of recurrence by approximately 50% in patients with locally advanced CRC harboring PI3K pathway mutations. Consequently, the NCCN 2025 guidelines now reference PI3K pathway mutations as a predictive biomarker for aspirin benefit. This clinical sensitivity is further supported by experimental evidence; aspirin has been shown to induce cell cycle arrest and promote apoptosis more effectively in *PIK3CA*-MT CRC cells than in wild-type counterparts, as reported by Gu et al.^14^ and Zumwalt et al.^15^.*PIK3CA* mutations are considered an indicator of poor prognosis owing to their association with resistance to chemotherapy^16,17^. In *PIK3CA*-MT cancers, including colorectal cancer, several insights into the rewiring of the glycolytic system and glutamine metabolism have been reported^18,19^, which indicates that the growth of *PIK3CA*-MT colorectal cancer could be inhibited by targeting glutamine metabolism^20^. Although we have previously established that glutamine depletion reduces the antitumor effects of aspirin in *PIK3CA*-MT colorectal cancer and that aspirin promotes glutamine metabolism^21^, the underlying mechanisms for the favorable response to aspirin in *PIK3CA*-MT colorectal cancer remain unclear.

When evaluating the antitumor effects of aspirin, it is essential to consider the tumor growth environment. Hypoxia, characterized by low oxygen levels, is a prominent feature of most solid cancers^22^. Increasing evidence suggests that hypoxia contributes to cancer therapy resistance by promoting chemoresistance, angiogenesis, and invasiveness^23,24^. Hypoxia-inducible factor-1α (HIF-1α), induced by hypoxia, plays important roles in the proliferation, apoptosis, angiogenesis, and stemness of cancer cells by upregulating cyclooxygenase-2 (COX-2) during hypoxia, thereby increasing the abundance of its enzyme product, prostaglandin E2 (PGE2)^25,26^. In addition to the well-known effect of aspirin on platelet activation, it has also been reported that most NSAIDs, including aspirin at doses above the COX-1 inhibitory anti-platelet threshold, have the capacity to inhibit COX-2-mediated prostacyclin production. This inhibition is thought to play a role in the mechanism associated with the hypoxia-induced angiogenic response in endothelial cells^27^. Although colorectal cancer induces a hypoxic microenvironment^28,29^, the molecular biological behavior of *PIK3CA*-MT colorectal cancer and the antitumor effects of aspirin under such conditions are not well understood. Therefore, we aimed to investigate the mechanisms through which hypoxia influences aspirin-associated intracellular glutamine accumulation in *PIK3CA*-MT colorectal cancer cell lines.

## Materials and methods

### Cell lines and culture

Human colon cancer cells, DLD-1, were sourced from the American Type Culture Collection. *PIK3CA* (H1047R/-) HCT116, *PIK3CA*-wild type (WT) HCT116, *PIK3CA* (E545K/+) SW48, and *PIK3CA*-WT SW48 were obtained from Horizon Discovery (Waterbeach, UK). Each cell line’s authenticity was verified through short tandem repeat profiling at the respective cell bank. Mycoplasma contamination was checked using the MycoAlert Mycoplasma Detection Kit (Lonza, Rockland, ME, USA), confirming their absence. Cells were used within three months post-thawing and cultured in RPMI 1640 medium (#01859-34, Nacalai Tesque, Kyoto, Japan) with 10% fetal bovine serum (#30264-85, Nacalai Tesque, Kyoto, Japan), 2 mM glutamine, 50 U/mL penicillin, and 100 µg/mL streptomycin (#26253-84, Nacalai Tesque, Kyoto, Japan). The culture medium was used within 2 weeks to prevent L-glutamine degradation. The cells were incubated at 37 °C in a humidified atmosphere with 5% CO2. Cells were incubated at 37 °C in a humidified atmosphere. Hypoxic conditions (1% O_2_ and 5% CO_2_) were established using a 9000EX CO_2_ incubator (Wakenyaku Co., Ltd.), with oxygen levels monitored and maintained according to the manufacturer’s instructions. For all experiments lasting up to 72 h, the culture medium was not exchanged to maintain stable experimental conditions. The pH of the medium was monitored using the phenol red indicator and remained within the physiological range (7.2–7.4) throughout the 72-hour period, ensuring that nutrient depletion or acidification did not confound the observed metabolic effects.

## Reagents

Aspirin, L-methionine sulfoximine (L-MS), and V-9302 were purchased from Sigma-Aldrich (#A5376-100G, Saint Louis, MO, USA), Nacalai Tesque (#21730-74, Kyoto, Japan), and Fujifilm (#1855871-76-9, Tokyo, Japan), respectively. For use, aspirin and L-MS were dissolved in dimethyl sulfoxide (DMSO, #13445-74, Nacalai Tesque, Kyoto, Japan) to prepare stock solutions of 1 M and 250 mM These were further diluted in the culture medium for experimental use, ensuring a final DMSO concentration of 0.2% across all treatment and control groups. V-9302 was dissolved in water to prepare a 20 mM stock solution, which was then diluted to the necessary working concentrations in the culture medium.

### Cell viability assay

Cell viability was determined using the Cell Counting Kit-8 assay (#347–07623, Dojindo, Kumamoto, Japan) according to the manufacturer’s guidelines. *PIK3CA*-MT HCT116 and *PIK3CA*-MT DLD-1 cells were plated in 96-well plates at a density of 2.5–5.0 × 10³ cells per well in 100 µL and incubated overnight. The next day, cells were exposed to 2 mM aspirin (ASA) and/or 20 µM V-9302 under 20% O_2_ (normoxic: NO) and 1% O_2_ (hypoxic: HY) conditions, with each drug diluted in a total volume of 200 µL. After the specified incubation period, 10 µ༬ WST-8 reagent was added to the medium and incubated for 4 h. Absorbance at 450 nm was measured using a SpectraMax iD5 (Molecular Devices, LLC, San Jose, CA, USA). Each independent biological experiment was performed in technical duplicates to ensure intra-experimental consistency. The results are expressed as the mean ± S.D. of independent biological replicates (*n*), as specified in the figure legends.

### Connectivity map

The Connectivity Map offers a public repository of over 7,000 gene expression profiles across 1,309 compounds (www.broad.mit.edu/cmap/)^30^. To evaluate gene expression variations, this study analyzed data from eight cell lines: A375 (malignant melanoma), A549 and HCC515 (lung cancer), HT-29 (*PIK3CA*-MT colorectal cancer), MCF-7 (*PIK3CA*-MT breast cancer), PC3 and VCaP (prostate cancer), and HA1E (immortalized kidney cells), all treated with 10 µM aspirin for 6 h. Gene enrichment analysis was performed using Metascape v3.5^31^(accessed on December 30, 2025) for genes with a score > 90.

### RNA sequencing and analysis

Total RNA was extracted from semi-confluent cells using the RNeasy Mini Kit (Qiagen). The RNA was then processed with the TruSeq Stranded mRNA Sample Prep Kit (Illumina, San Diego, CA, USA). Poly(A) RNA libraries were prepared using the TruSeq Stranded mRNA Library Preparation Kit (Illumina), followed by sequencing as 100-bp paired-end reads on the Illumina NovaSeq6000 platform. RNA-seq reads were quantified using ikra v1.2.2^32^, an RNA-seq pipeline centered on Salmon^33^(accessed on April 30, 2020). The ikra pipeline automated the RNA-seq data analysis process, including read quality control (Trim Galore version 0.6.6 (Babraham Bioinformatics, UK^34^ with Cutadapt^35^ and transcript quantification (Salmon version 1.4.1 using reference transcript sets in GENCODE release 31 for humans). The default settings were applied for all tools in the ikra pipeline. The count tables were imported into iDEP v2.01 (accessed on December 30, 2025), a web-based tool used for pathway and differential expression analyses of RNA-seq data^36^ The transcript reads were filtered to retain only those with a minimum of 0.5 counts per million (CPM) in at least one sample. The data underwent transformation using EdgeR with a log2 (CPM + 4) calculation, where a pseudocount of 4 was applied. Gene set enrichment analysis (GSEA)^37^ was performed within the iDEP software, utilizing fold-change values derived from DESeq2. Gene sets with a false discovery rate (FDR) q-value of less than 0.05 were selected for further exploration.

### Metabolite analysis

HCT116 (*PIK3CA*-MT/WT) cells were cultured in 100 mm dishes with culture medium overnight. Cells were then treated with DMSO, aspirin (2 mM), and V-9302 (20 µM) or L-MS (10 mM) with aspirin (2 mM) divided into groups under 1% O_2_ and 20% O_2_ conditions, followed by a 24-h incubation period. The cells were grown to sub-confluence, harvested with trypsin (#35553-74, Nacalai Tesque, Kyoto, Japan), and suspended in phosphate-buffered saline (PBS, #14249-95, Nacalai Tesque, Kyoto, Japan). The extraction and analysis of the metabolites were conducted as previously described^38^. In brief, the cells were centrifuged at 1,000 × g for 5 min at 4 °C, and the resulting pellets were resuspended in 200 µL of methanol. The suspension was mixed with Milli-Q water and chloroform in a 5:2:5 volume ratio, followed by centrifugation at 9,000 × g for 15 min at 4 °C. The aqueous phase was then filtered through a 3 kDa cutoff filter (Amicon Ultra, Merck KGaA) to remove high molecular weight components. The filtrate was concentrated by centrifugation, and metabolites corresponding to 5 × 10⁵ cells were dissolved in 5 µL of ammonium hydroxide before being analyzed by liquid chromatography coupled with tandem mass spectrometry (LC-MS/MS). Chromatographic separation was carried out using an UltiMate 3000 HPLC system (Thermo Fisher Scientific, Rockford, IL, USA) fitted with a YMC-Triart PFP column (3 mm particle size, 12 nm pore size, 150 × 2.1 mm; YMC Co., Ltd., Kyoto, Japan). The mobile phase consisted of 0.5% formic acid in water, with a flow rate of 0.2 mL/min and an injection volume of 5 µL. The HPLC system was connected to a Thermo Scientific TSQ Endura Triple Quadrupole Mass Spectrometer (Thermo Fisher Scientific). Electrospray ionization was applied in positive mode for the detection of glutamine and glutamic acid, and in negative mode for other tricarboxylic acid (TCA) cycle metabolites. The spray voltage was set to 3,500 V in positive mode and 2,500 V in negative mode. TCA cycle metabolites were detected using multiple reaction monitoring (MRM) in tandem mass spectrometry. A summary of the MS parameters is shown in Table S1. To ensure analytical precision and monitor ion suppression, internal standards (IS) were added to each sample during extraction. TCA standard stock solutions were prepared at a 50 mM concentration in a solution of ammonium hydroxide. All stock solutions were prepared daily just before running samples in the LC-MS/MS system. Calibration curves of TCA standard were prepared from the working stock solution at concentrations of 100 nM – 5 mM. Quality control (QC) samples were prepared identically to the standards at final concentrations of 5 and 500 µM and 5 mM. The relative standard deviation (RSD) for all quantified metabolites was maintained at less than 15%, and solvent blanks were injected between samples to rule out carryover. Metabolites were reported as absolute concentrations normalized to cell numbers.

### ROS-Glo™ H2O2 assay

Oxidative stress was evaluated using the ROS-Glo™ H2O2 Assay (Promega, Madison, WI, USA) according to the manufacturer’s instructions. This bioluminescent assay specifically measures hydrogen peroxide (H_2_O_2_), a reactive oxygen species (ROS), in cell cultures or enzyme reactions. In brief, 1.5 × 10⁴ cells were seeded into 96-well plates and cultured in RPMI 1640 medium. The cells were treated with 2 mM aspirin, 20 µM V-9302, or 10 mM L-MS and incubated at 37 °C with 5% CO_2_ for 24 h. Following incubation, the H_2_O_2_ substrate solution was added for 2 h to produce a luciferin precursor. The ROS-Glo™ Detection Solution was then added for 20 min, converting the precursor into luciferin and activating Ultra-Glo™ Recombinant Luciferase, generating a light signal proportional to the H_2_O_2_ concentration. Bioluminescence was measured using the EnSpire™ 2300 Multilabel Reader. and the results were expressed in relative luminescence units. For ROS quantification, cells were seeded at an identical density across all groups at the start of the experiment. Therefore, ROS levels are reported as raw RLU to represent the total cumulative oxidative burden generated by the treatments within the niche. Normalization by post-treatment cell counts was intentionally avoided to prevent the mathematical masking of the total oxidative stress that drives the subsequent biological outcomes. Each independent biological experiment was performed in technical duplicates to ensure intra-experimental consistency. Data are expressed as the mean ± S.D. of three independent biological replicates (*n* = 3), as specified in the figure legends.

### Colony formation assay

We seeded *PIK3CA*-MT HCT116 colorectal cancer cells at a density of 2,000 cells per well in 6-well plates and allowed them to grow for 24 h. The cells were then exposed to 2 mM aspirin (ASA) and/or 20 µM V-9302 under both 20% O_2_ (NO) and 1% O_2_ (HY) conditions, and incubated for 6 days. After incubation, the cells were fixed using 10% formalin (Fujifilm, Tokyo, Japan) and stained with 0.1% crystal violet (Sigma-Aldrich, Saint Louis, MO, USA). The total area and percentage of area covered by colonies were quantified using ImageJ software (version 1.54, National Institutes of Health, USA). Digital images of the culture plates were first converted to 8-bit grayscale to facilitate thresholding. To eliminate non-specific background signals and uneven illumination, the Subtract Background command was applied with a rolling ball radius of 50 pixels. The colony regions were defined by applying an automated thresholding algorithm (Otsu method). Following binarization, the Analyze Particles function was utilized to measure the total area. To ensure the exclusion of minor artifacts and cellular debris, a size-based filter was applied, including only particles larger than 100 pixels. The results were expressed as the “Area Fraction” (%), representing the ratio of the colony-occupied area to the total surface area of the well. Each independent biological experiment was performed in technical duplicates to ensure intra-experimental consistency. The results are expressed as the mean ± S.D. of independent biological replicates (*n*), as specified in the figure legends.

### Statistical analysis

Data are presented as the mean ± standard deviation (S.D.) of independent biological replicates (*n*). For cell viability, colony formation, and ROS-Glo assays, each independent biological experiment was performed in technical duplicates to ensure intra-experimental consistency. Differences among three or more groups were assessed using one-way analysis of variance (ANOVA) followed by Tukey’s post-hoc tests for multiple comparisons. Two-group comparisons were conducted using the two-tailed unpaired Student’s t-test. Exact *P*-values are indicated in the figures to ensure full transparency, and a *P*-value < 0.05 was considered statistically significant relative to the specified control group. Statistical analyses and data visualization were performed using GraphPad Prism 9 (GraphPad Software Inc., San Diego, CA, USA).

## Results

### Bioinformatic analysis suggest aspirin influences the metabolism of amino acids and derivatives and HIF-1 signaling pathway in *PIK3CA*-MT HT-29 colon cancer cells

**Fig. 1:**
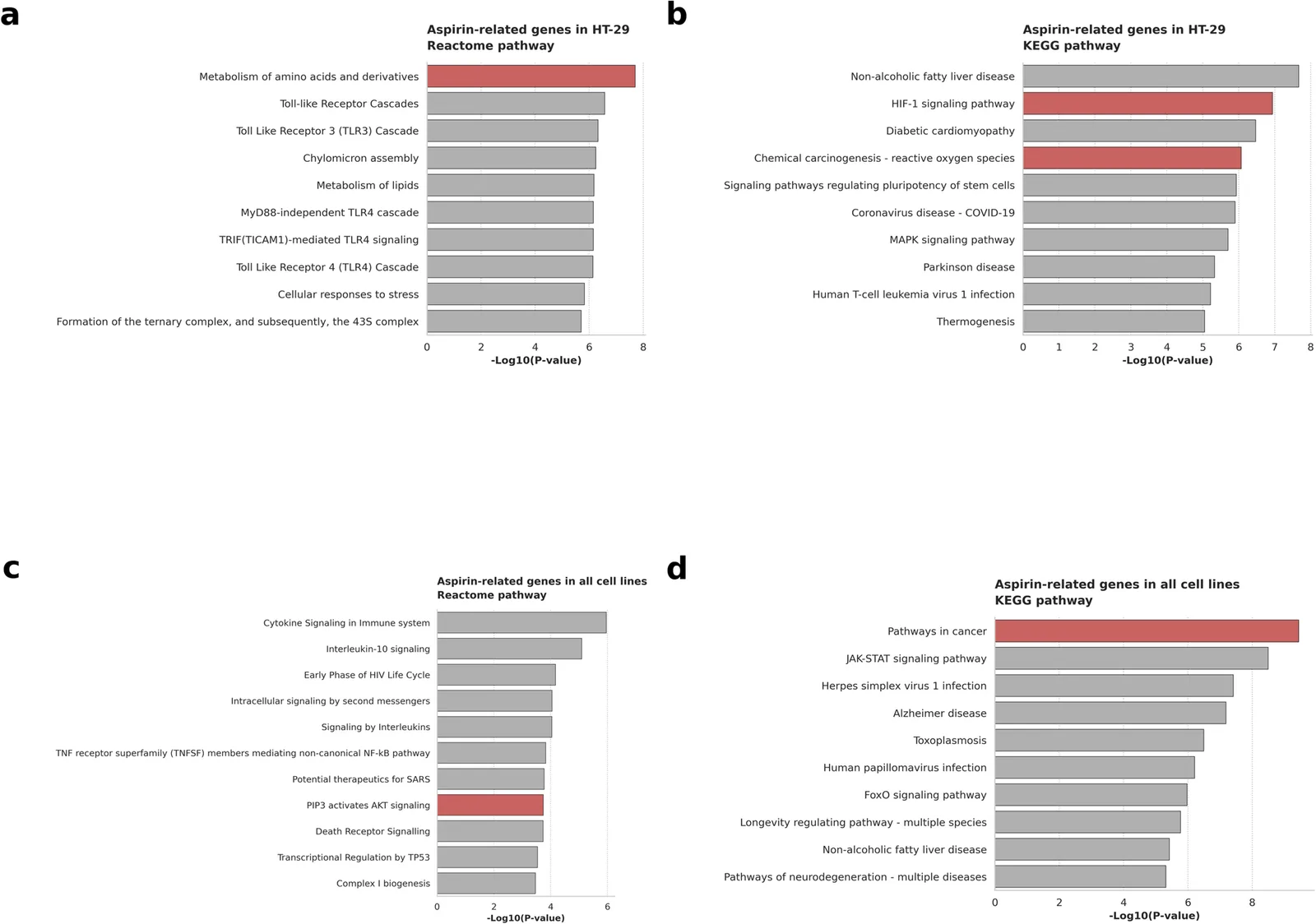
Enrichment analysis of aspirin-related genes across multiple cell lines and *PIK3CA*-mutated colon cancer cells. Enrichment profiles were generated based on Connectivity Map (CMap) data. (**A**,** B**) Metascape enrichment analysis of “aspirin-related genes” in HT-29 (*PIK3CA*-MT) cells, highlighting pathways related to amino acid metabolism and hypoxia. (**C**,** D**) Comparative enrichment analysis across eight reference cell lines, showing a lack of similar metabolic signatures. Dot size and color reflect the significance and magnitude of enrichment, respectively.

We attempted to estimate the mechanism of action of aspirin by using Connectivity Map to perform an enrichment analysis of genes that cause gene expression changes similar to those caused by aspirin treatment. We performed enrichment analysis on “aspirin-related genes” (similarity score > 90, Table S2A–C) using Metascape, and the results is possible that aspirin influenced the metabolism of amino acids and their derivatives and hypoxic pathways in the *PIK3CA*-MT HT-29 colorectal cancer cell line (Fig. 1A, B). However, this was not observed in the analysis of the eight cell lines (Fig. 1C, D) nor in that of MCF-7 (Fig. S1). Particularly in colorectal cancer cells, a significant impact of *PIK3CA* mutations on amino acid metabolism was possible. These bioinformatic analysis were utilized as a supportive, hypothesis-generating tool, suggesting that the metabolic impact of aspirin may be modulated by the genetic and metabolic background of each cell type.

### Hypoxia induces amino acid import in *PIK3CA*-MT, but not *PIK3CA*-WT, SW48 colorectal cancer cells

**Fig. 2:**
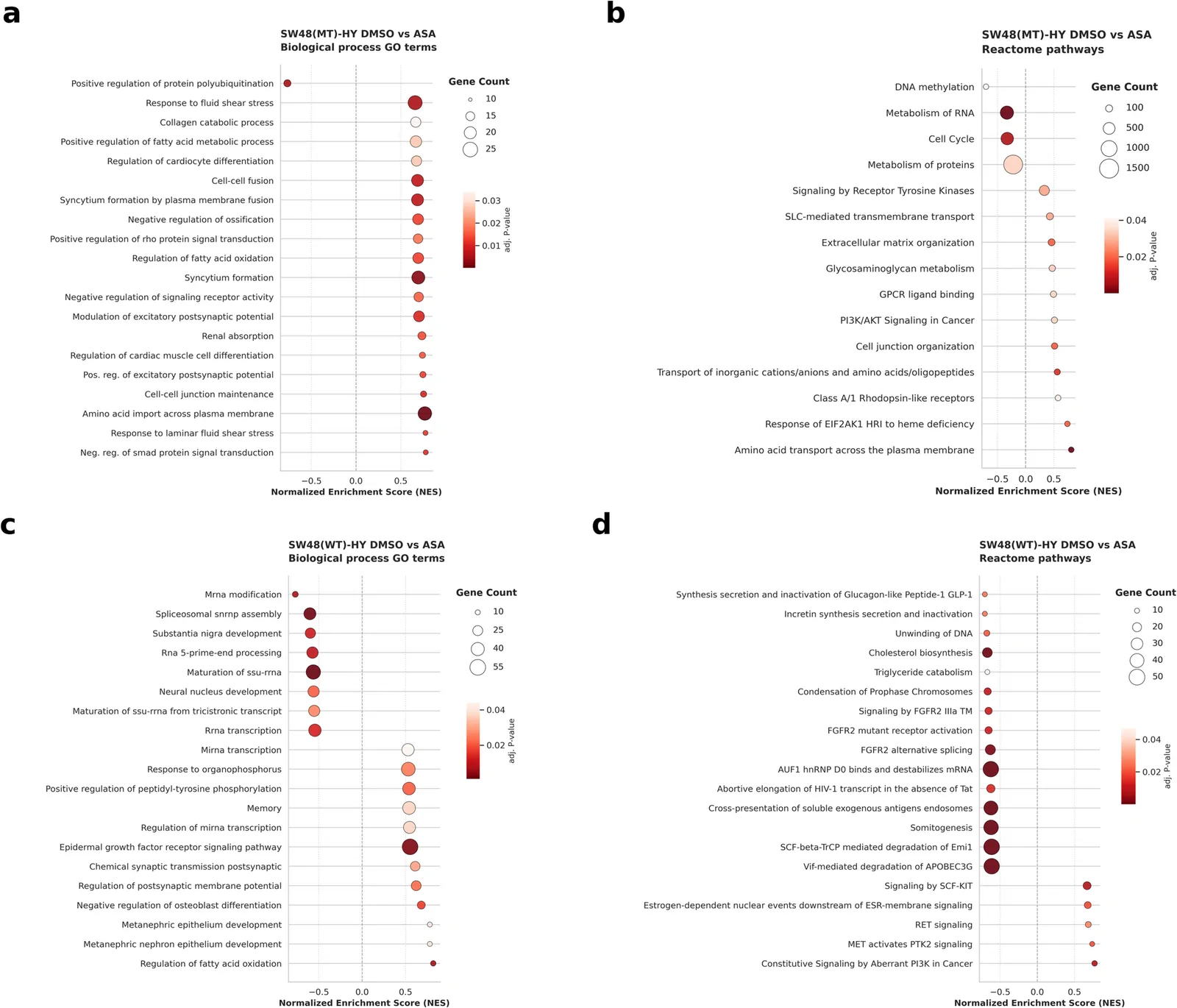
Aspirin preferentially upregulates amino acid transport pathways in *PIK3CA*-mutated colorectal cancer cells under hypoxia. (A, B) Gene Set Enrichment Analysis (GSEA) results showing significant enrichment of “Amino acid import/transport” related pathways in *PIK3CA*-mutated (MT) SW48 cells treated with aspirin versus DMSO under hypoxic conditions (1% O_2_). Analysis was performed using the (**A**) Gene Ontology (GO) Biological Process and (**B**) Reactome databases (FDR q < 0.05). (C, D) Corresponding GSEA plots for *PIK3CA*-wild type (WT) SW48 cells, demonstrating a lack of significant enrichment for amino acid transport pathways in either (**C**) GO or (**D**) Reactome databases. In the bubble plots, dot size represents the gene count, and the color gradient indicates the adjusted *P*-value.

Guided by the analysis of public gene expression profiles across various cell lines, which suggested a potential involvement of aspirin in amino acid metabolism and hypoxic signaling particularly in HT-29 cells, we sought to examine whether these associations were linked to *PIK3CA* mutation status. To this end, we compared aspirin-induced gene expression patterns between parental SW48 colorectal cancer cells (*PIK3CA*-WT) and their isogenic *PIK3CA*-mutant (*PIK3CA*-MT) counterpart. In *PIK3CA*-MT SW48 cells, GSEA utilizing both Gene Ontology (GO) and Reactome databases revealed significant enrichment of genes associated with amino acid transport pathways in the aspirin-treated group compared to the DMSO-treated group under hypoxic conditions (FDR *q* < 0.05; Fig. 2A, B). In contrast, these pathways were not significantly upregulated in aspirin-treated *PIK3CA*-WT SW48 cells (Fig. 2C, D). Although the fold changes of individual genes were modest, the consistent enrichment of these pathways across different databases and the stricter statistical threshold underscore the biological significance of aspirin’s impact on amino acid metabolism in *PIK3CA*-mutated cells.

### Intracellular glutamine level rises in *PIK3CA*-MT HCT116 colorectal cancer cells following aspirin administration and under hypoxic conditions

**Fig. 3:**
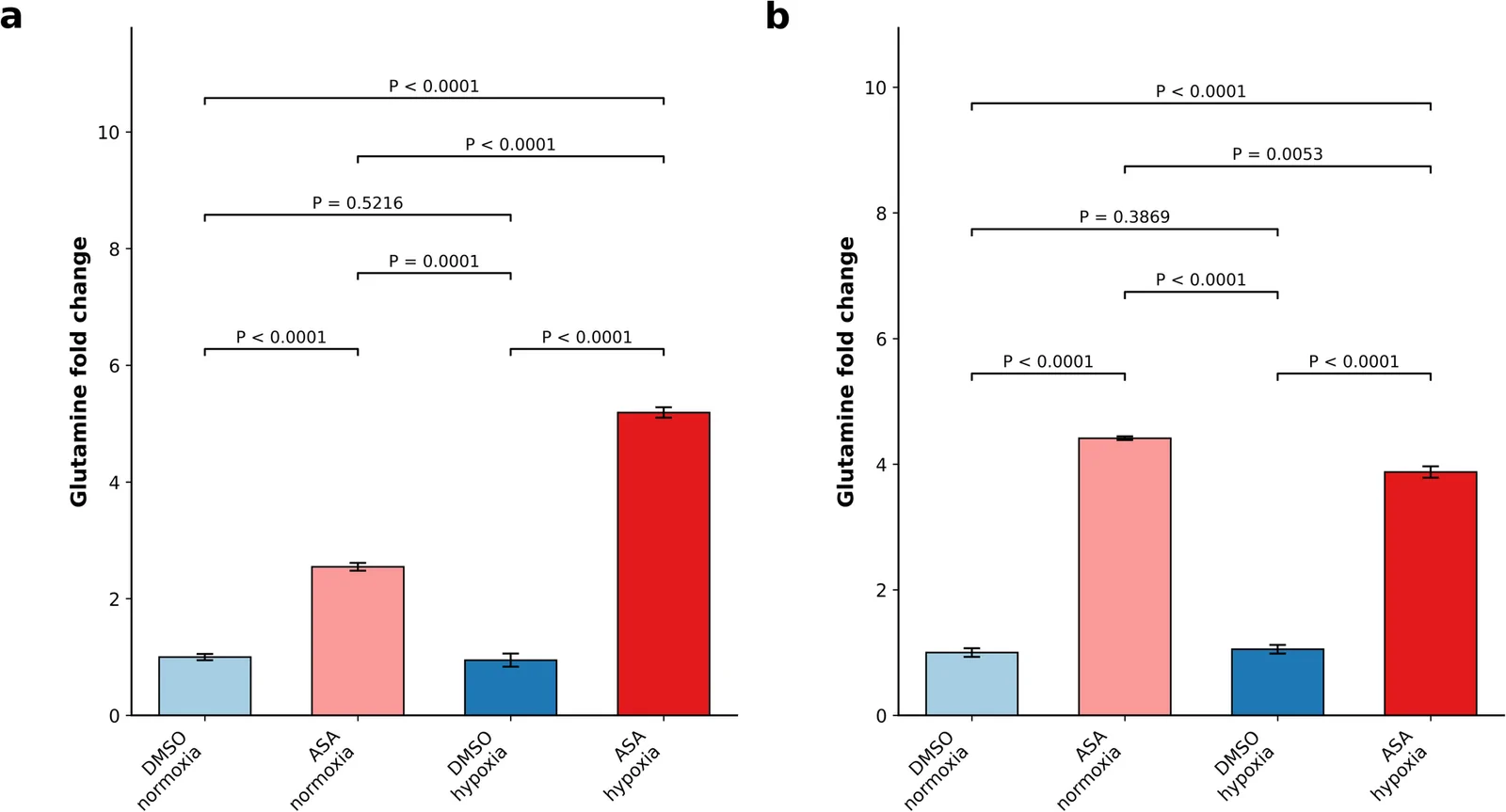
Aspirin-induced alterations in intracellular glutamine levels in *PIK3CA*-mutated colorectal cancer cells. Intracellular glutamine levels were quantified using LC-MS/MS in (**A**) *PIK3CA*-mutant (MT) and (**B**) *PIK3CA*-wild-type (WT) HCT116 colorectal cancer cells. All values were normalized to the DMSO-treated controls under normoxia, which were set to 1.0. Data represent the mean ± S.D. (*n* = 3 independent biological replicates). Statistical significance was determined using one-way ANOVA followed by Tukey’s post-hoc test.

Given the observed link between aspirin treatment and amino acid transporter expression under hypoxia in *PIK3CA*-mutant cells, we next explored whether these changes are accompanied by alterations in intracellular glutamine metabolism. We employed a mass spectrometry-based metabolomics approach to quantify TCA cycle-related metabolites in *PIK3CA*-MT colorectal cancer cells. Among these metabolites, glutamine was most strongly affected by *PIK3CA* mutation status, aspirin treatment, and hypoxic conditions in HCT116 colorectal cancer cells (Fig. S2A, B). *PIK3CA*-MT HCT116 cells exhibited relatively increased expression of genes involved in amino acid uptake in response to aspirin in hypoxia, compared with wild-type cells (Fig. S3A-C). Aspirin treatment significantly increased intracellular glutamine levels in HCT116 cells, regardless of oxygen availability and *PIK3CA* mutation status. Notably, under hypoxic conditions, aspirin treatment led to a further increase in intracellular glutamine levels in *PIK3CA*-MT HCT116 cells, whereas no such additive effect was observed in *PIK3CA*-WT cells (Fig. 3A, B). These results indicate that hypoxia additively enhances the aspirin-induced increase in intracellular glutamine level, with a more pronounced effect observed in *PIK3CA*-MT colorectal cancer cells.

### *PIK3CA*-MT HCT116 colorectal cancer cells exhibit a decrease in intracellular glutamine levels and increase in ROS levels upon treatment with glutamine-targeted drugs

**Fig. 4:**
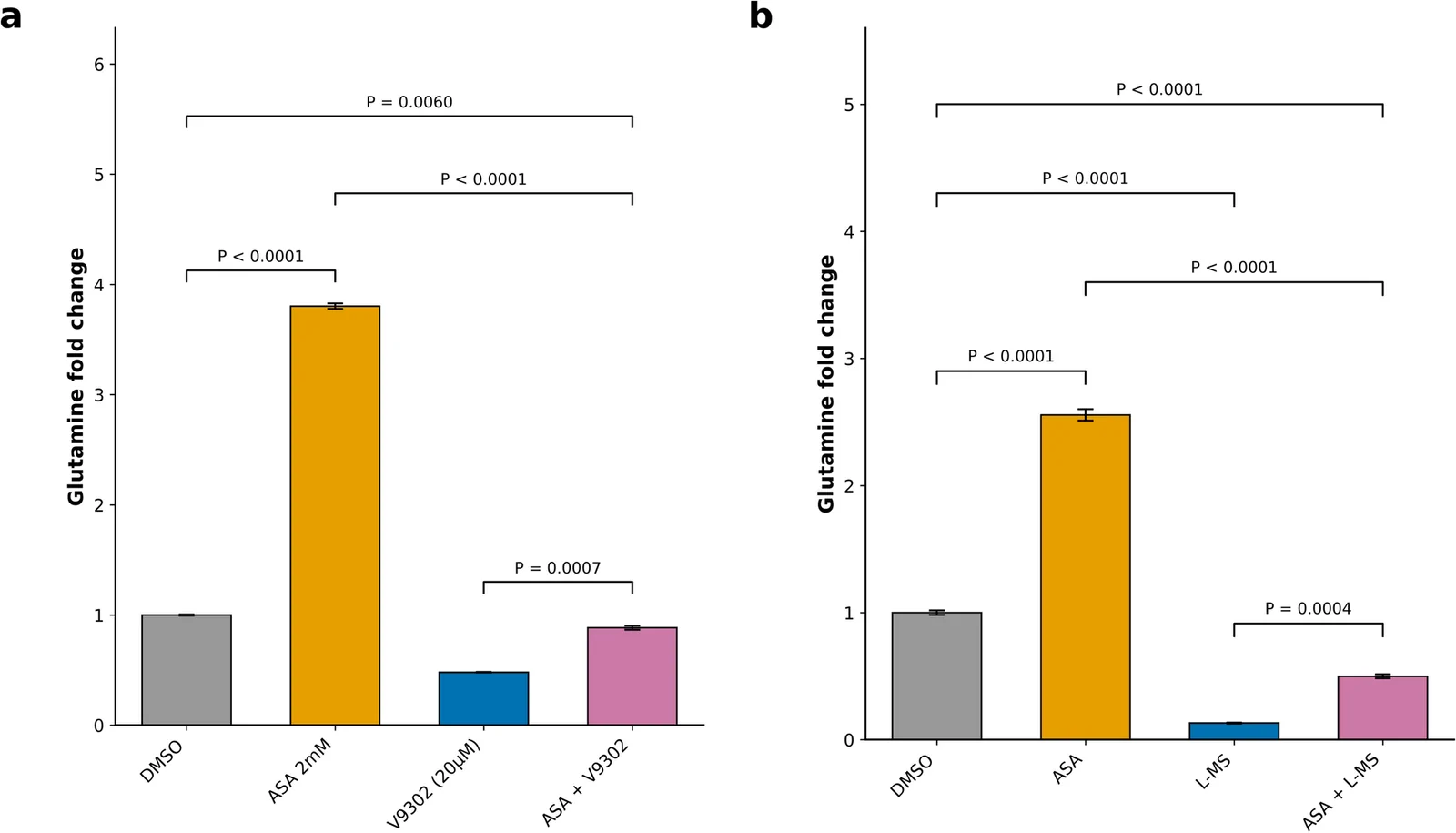
Impact of glutamine-targeted drugs on intracellular glutamine levels in *PIK3CA*-mutated colorectal cancer cells under hypoxia. Intracellular glutamine levels were quantified via LC-MS/MS in *PIK3CA*-MT HCT116 cells following treatment with (**A**) V-9302 (20 µM) or (**B**) L-MS (10 mM) in the presence or absence of aspirin (2 mM) under hypoxic (1% O_2_) conditions. All values were normalized to the DMSO-treated control (set to 1.0). Data represent the mean ± S.D. (*n* = 3 independent biological replicates). Statistical significance was determined using one-way ANOVA followed by Tukey’s post-hoc test.

We further investigated the mechanism underlying the aspirin-induced elevation of intracellular glutamine levels in *PIK3CA*-MT colon cancer cells under hypoxia. We used L-MS, a glutamine synthetase inhibitor, and V-9302, a glutamine transporter ASCT2 inhibitor, to examine the regulation of glutamine dynamics in response to aspirin treatment (Fig. S4). In *PIK3CA*-MT HCT116 colorectal cancer cells, intracellular glutamine levels decreased following treatment with L-MS and V-9302. Moreover, both drugs counteracted the increase in intracellular glutamine levels induced by aspirin (Fig. 4A, B). These findings suggest that the aspirin-induced increase in intracellular glutamine levels is regulated at both the levels of de novo synthesis and import from the extracellular environment. Glutaminolysis promotes nicotinamide adenine dinucleotide phosphate (NADPH) generation through glutamate dehydrogenase (GLUD)-mediated glutamate degradation, thereby contributing to ROS homeostasis^39^. We observed that ROS levels were increased following exposure to aspirin in *PIK3CA*-MT HCT116 cells. Of note, co-treatment with aspirin and either V-9302 or L-MS resulted in a more pronounced increase in intracellular ROS levels (Fig. S5A, B), revealing a phenomenological association between increased intracellular ROS levels and reduced cell viability upon co-treatment. While these findings suggest that oxidative stress may accompany the growth-inhibitory effects, the definitive causal role remains to be elucidated.

### V-9302 reduces the growth of *PIK3CA*-MT colorectal cancer cells when combined with aspirin

**Fig. 5:**
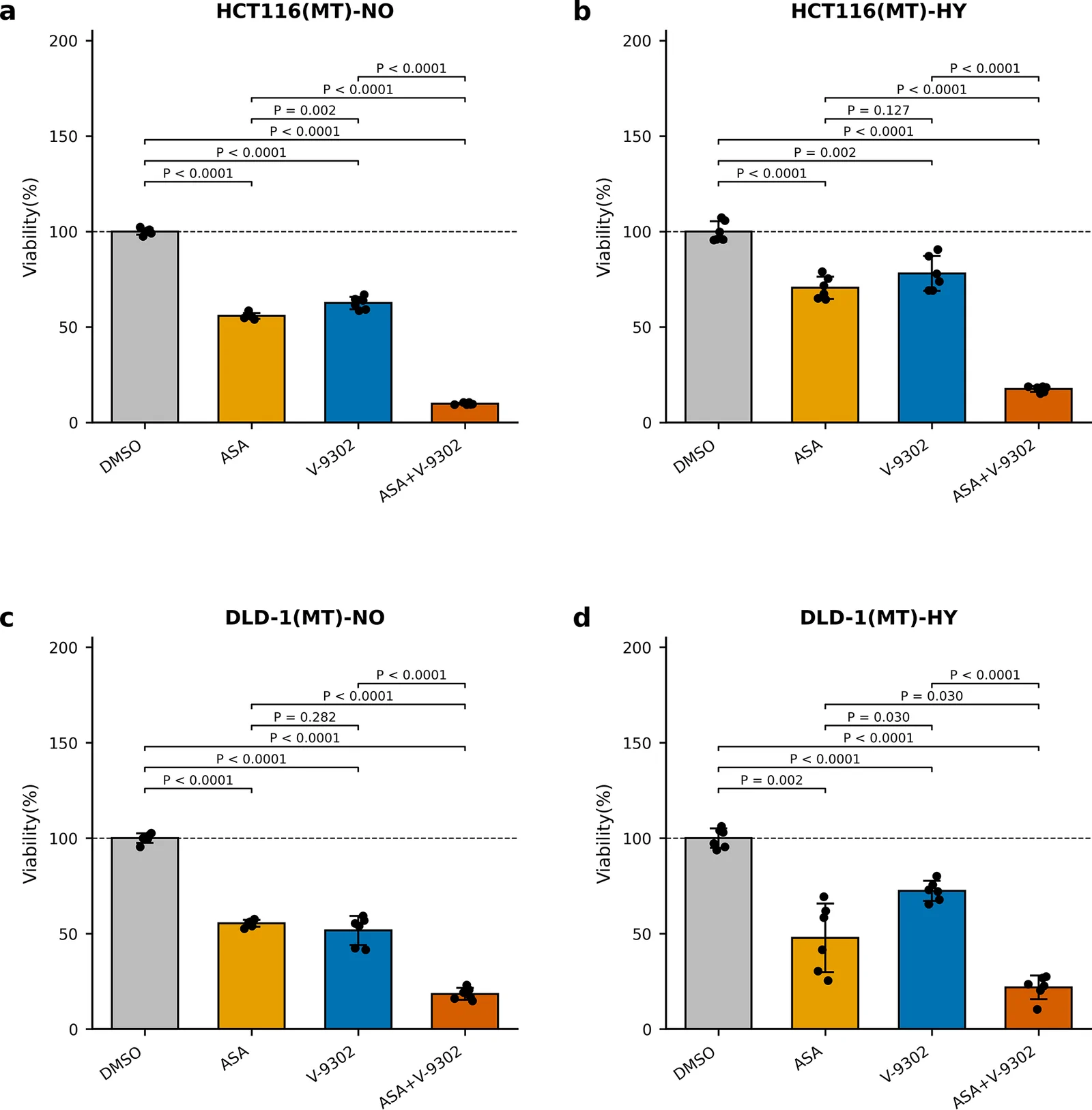
Combinatorial effects of aspirin and V-9302 on the viability of *PIK3CA*-mutant colorectal cancer cells. Cell viability was assessed using the CCK-8 assay after 72 h of incubation with 2 mM aspirin (ASA) and/or 20 µM V-9302. (**A**, **B**) *PIK3CA*-mutant (MT) HCT116 cells and (**C**, **D**) *PIK3CA*-MT DLD-1 cells were evaluated under (**A**, **C**) normoxic (20% O_2_) and (**B**, **D**) hypoxic (1% O_2_) conditions. Each independent biological experiment was performed in technical duplicates to ensure intra-experimental consistency. Results are expressed as the mean ± S.D. (*n* = 3 independent biological replicates). Statistical significance was determined using one-way ANOVA followed by Tukey’s post-hoc test.

**Fig. 6:**
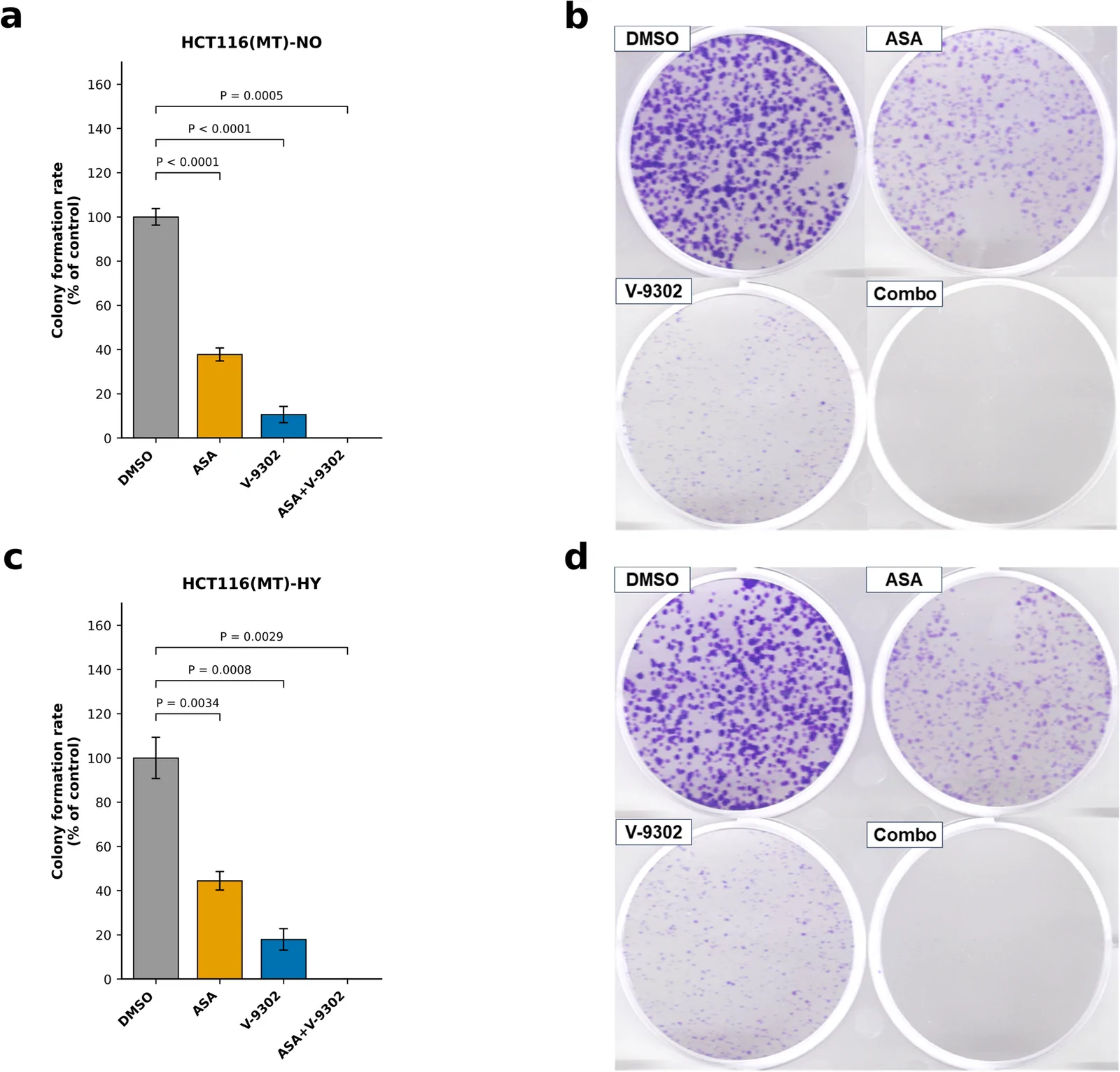
Aspirin and V-9302 exhibit synergistic inhibitory effects on long-term colony formation. (A, C) Quantitative analysis of colony formation in *PIK3CA*-MT HCT116 cells treated with ASA (2 mM) and V-9302 (20 µM) under (**A**) normoxic and (**C**) hypoxic conditions. Colony area fractions were calculated as a percentage relative to the DMSO-treated control. (B, D) Representative images of crystal violet-stained colonies under (**B**) normoxia and (**D**) hypoxia are shown. All assays were performed in technical duplicates for each biological replicate. Data represent the mean ± S.D. (*n* = 3 independent biological replicates). Statistical analyses were performed using a two-tailed Student’s t-test.

The alteration of glutamine metabolism is reportedly a novel strategy for cancer therapy in glutamine-dependent hematologic and solid tumors, including *PIK3CA*-MT colorectal cancer^40–42^. We treated *PIK3CA*-MT colorectal cancer cells with aspirin in combination with V-9302 under normoxic (20% O_2_, NO) and hypoxic (1% O_2_, HY) conditions. The combination of aspirin and V-9302, under both normoxic and hypoxic conditions, resulted in a more pronounced inhibition of cell growth compared to the single treatment in both *PIK3CA*-MT HCT116 and *PIK3CA*-MT DLD-1 colorectal cancer cells (Fig. 5A–D). Moreover, the colony formation assay on HCT116 cells confirmed the combinatorial antitumor effect (Fig. 6A-D). However, the combination of aspirin and L-MS did not show any inhibitory effect on cell proliferation, compared with L-MS alone (Fig. S6). These results suggested that the inhibition of transporters involved in the glutamine pathway enhanced the efficacy of aspirin in *PIK3CA*-MT colorectal cancer cells.

## Discussion

We previously reported that glutamine is essential for the growth of *PIK3CA*-MT colorectal cancer cells and that the inhibitory effect of aspirin on growth is dependent on the presence of glutamine^21^. In this study, we focused on investigating the mechanisms through which aspirin modulates the behavior of glutamine under hypoxic conditions prevalent in the tumor microenvironment. The results demonstrated that aspirin exerted an antitumor effect even under hypoxic conditions in *PIK3CA*-MT colorectal cancer cells and that its combination with drugs that reduced intracellular glutamine levels enhanced its antitumor effects.

On average, the oxygen level within tumors is approximately 1–2% lower than that in normal tissue^43^. The overexpression of HIF-1α, a master regulator involved in cellular adaptation to hypoxia, has been detected in both primary and metastatic colorectal cancer lesions^44^. Intratumor heterogeneity and mutations in *TP53*, *APC*, *KRAS*, and *PIK3CA* are positively associated with the hyperactivation of hypoxia signaling^45,46^. The clinical significance of *PIK3CA* mutations as a predictive biomarker for aspirin efficacy has been a subject of extensive debate. Recent results from the ALASCCA study (Martling et al., 2025) and updated NCCN guidelines suggest that the underlying metabolic context is crucial for identifying responsive subgroup^13^. Our study provides a mechanistic basis for these observations, showing that the synergistic effect of aspirin and hypoxia is mediated through alterations in glutamine metabolism.

Glutamine plays a crucial role in the growth and survival of various hematological and solid tumors^47,48^. Under normal conditions, glucose is converted to acetyl-CoA, which enters the TCA cycle. However, cancer cells exhibit the Warburg effect, converting glucose to lactate even in the presence of oxygen, and they rely on glutamine to replenish the TCA cycle^49^. This process provides the necessary energy for cell proliferation and regulates oxidative phosphorylation, nucleotide biosynthesis, redox homeostasis, and ROS homeostasis. As such, glutamine contributes actively to tumor growth and survival, particularly under hypoxic conditions^50^.

Oxidative stress, characterized by an imbalance between ROS and antioxidants, has been linked to cancer and various other pathologies^51^. ROS can exert paradoxical effects on cancer evolution, either initiating/stimulating tumorigenesis and supporting the transformation/proliferation of cancer cells or inducing cell death^52^. In our study, we confirmed alterations in ROS balance induced by aspirin, V-9302, and L-MS (Fig. 5C, D). While there have been conflicting reports regarding ROS generation in cells treated with NSAIDs^53^, colon cancer cells reportedly exhibit stronger ROS generation than other cancer cell types^54,55^, which is consistent with the findings of our present study. Precursors such as glutamate and cysteine are directly involved in the regulation of ROS homeostasis by supplying glutathione synthesis and promoting the production of NADPH through GLUD^39^. We believe that this mechanism may explain the increased ROS levels observed in cells exposed to V-9302 (Fig. 5C). L-MS, a glutamine synthetase inhibitor, did not increase ROS levels when used alone; however, it increased ROS levels when used in combination with aspirin (Fig. 5D). Regarding ROS assessment, we focused on the total oxidative phenomenon rather than per-cell production rates. Since initial seeding was uniform, the observed increase in total RLU reflects the net accumulation of ROS, which we believe is the primary driver of the anti-tumor response. Normalization by post-treatment cell counts was intentionally avoided to prevent the mathematical masking of the total oxidative stress that drives cell death. Since antioxidant rescue experiments were not performed, the direct causality between ROS and anti-tumor efficacy remains a subject for future investigation.

Our study demonstrated that intracellular glutamine levels were increased by aspirin, especially under hypoxic conditions (Fig. 4). *PIK3CA*-MT colorectal cancer relies on glutamine for growth and survival^21^, and elevated intracellular glutamine may contribute to drug resistance. These findings can be reconciled with recent reports by Holt et al. (2023), who demonstrated using isotope tracing that while aspirin decreased glutaminolysis, it paradoxically led to an accumulation of intracellular glutamine if downstream utilization was more severely impaired than uptake^56^. Our static metabolomic data reflect this net balance, suggesting that under metabolic stress, uptake may outweigh utilization. Therefore, we propose that combining drugs that reduce intracellular glutamine levels with aspirin may enhance the antitumor effect of aspirin. Our results indicated that L-MS had no effect on cell proliferation with or without hypoxia, but V-9302 influenced cell proliferation, particularly in combination with aspirin (Fig. 6).

V-9302 is a competitive small molecule antagonist that selectively and potently targets transmembrane glutamine flux by specifically targeting the amino acid transporter ASCT2 (*SLC1A5*)^41^. Given the critical role of glutamine in cancer cell growth and homeostasis, novel therapies targeting glutamine metabolism have shown potential anticancer properties. However, current efforts in this field have encountered limited success^40,42^. One strategy currently being evaluated in early-phase clinical trials focuses on mitochondrial glutaminase (GLS1), an enzyme responsible for converting glutamine to glutamate (e.g., CB-839 from Calithera Biosciences)^40^. GLS1 levels reportedly increase following mTOR inhibition^57,58^, and we have previously proposed the combination of a GLS inhibitor and aspirin, an mTOR inhibitor, for the treatment of *PIK3CA*-MT colorectal cancer. Although promising, this strategy has limitations as it does not fully address the extramitochondrial roles of glutamine, which include the RAS-independent activation of mitogen-activated protein kinase signaling^59^.

We hypothesize that inhibiting intracellular glutamine transport via ASCT2, such as with V-9302, may be a more effective strategy for targeting glutamine metabolism in *PIK3CA*-mutant cancer cells than targeting downstream enzymatic activities, such as GLS1 or glutamine synthetase. While L-MS inhibits de novo glutamine synthesis from glutamate, it does not block the uptake of glutamine from the extracellular environment. In our study, L-MS treatment had a limited impact on cell proliferation, whereas V-9302 significantly enhanced the antitumor effects of aspirin. These findings suggest that when subjected to metabolic stress—induced by the combination of aspirin and hypoxia—*PIK3CA*-mutant colorectal cancer cells appear to depend predominantly on extracellular glutamine uptake rather than de novo synthesis to maintain intracellular glutamine levels, as evidenced by their high sensitivity to V-9302.

This study has several limitations that should be acknowledged. First, we could not assess the mechanisms through which additional mutational backgrounds, particularly in the context of *PIK3CA* mutations, or the immune environment may alter the effects of aspirin. *PIK3CA* mutations are associated with the expression of immune-related genes^60^, and therefore, it is essential to also investigate the impact of aspirin on immune cells. Additionally, Kerk et al. reported that RAS promotes glutamine metabolism, highlighting the importance of assessing co-mutations with *PIK3CA* mutations in future research^61^. Second, while V-9302 is reported to be highly selective, we cannot completely exclude the possibility of potential off-target effects inherent to small-molecule inhibitors. Our conclusions regarding ASCT2-specific causality should be viewed as pharmacological observations that warrant further validation using genetic approaches for gene silencing, such as siRNA- or CRISPR-based methods to definitively confirm the specificity of these findings.

Third, our findings regarding the potential upregulation of glutamine transporters rely primarily on mRNA-level data. While we attempted protein-level validation for ASCT2 via Western blotting, the protein was detected as a diffuse smear likely due to N-glycosylation. This technical limitation hindered precise densitometric quantification, but we believe that the significant alterations in actual intracellular glutamine accumulation provide a compelling functional basis for our conclusions.

Fourth, regarding our ROS analysis, we acknowledge the limitation of providing raw RLU values rather than per-cell production rates. Furthermore, as antioxidant rescue experiments were not performed in this study, the observed ROS accumulation remains an associated phenomenon rather than a proven causal mediator of cell death. Establishing a direct causal link between this metabolic stress and anti-tumor efficacy remains a subject for future investigation.

Regarding our experimental conditions, the concentration of aspirin used in this study (2 mM) exceeds the physiological levels typically achieved in human plasma. However, this concentration is consistent with previous in vitro mechanistic studies and was necessary to capture acute metabolic changes. Future studies using more physiologically relevant models or long-term low-dose exposure are warranted to further bridge these findings to clinical settings.

Overall, our findings suggest that aspirin may enhance metabolic vulnerability to glutamine uptake inhibition, providing a promising rationale for combining aspirin with agents targeting glutamine transport. However, a significant limitation of the present study is that our observations were conducted exclusively using in vitro culture systems. The in vivo tumor microenvironment involves complex interactions between cancer cells, stromal components, and the immune system, alongside fluctuating nutrient and oxygen gradients that were not fully captured in our models. Therefore, further in vivo investigations, including studies using mouse xenograft models or patient-derived xenografts (PDX) of *PIK3CA*-mutated colorectal cancer, are essential to validate the efficacy and safety of this combinatorial approach. Such validation will be a critical step toward translating these metabolic strategies into effective clinical applications for patients with *PIK3CA*-mutated colorectal cancer.

## Conclusions

Targeted metabolomics demonstrated that aspirin treatment and hypoxic stimulation additively upregulated intracellular glutamine levels in *PIK3CA*-MT colorectal cancer cells. This finding suggests the potential for hypoxia to inhibit the effectiveness of aspirin against *PIK3CA*-MT colorectal cancer. Additionally, our findings highlight that the antitumor efficacy of aspirin can be enhanced by targeting glutamine in *PIK3CA*-MT colorectal cancer.

## Supplementary Information

Below is the link to the electronic supplementary material.


Supplementary Material 1



Supplementary Material 2



Supplementary Material 3



Supplementary Material 4



Supplementary Material 5



Supplementary Material 6



Supplementary Material 7



Supplementary Material 8



Supplementary Material 9



Supplementary Material 10


## Data Availability

The RNA-seq data generated in this study have been deposited in the DNA Data Bank of Japan (DDBJ) Sequence Read Archive under accession numbers DRR493298–DRR493309, BioProject ID PRJDB16166. The data are publicly available at the DDBJ database (https://www.ddbj.nig.ac.jp/). The targeted metabolomics data, including raw mass spectrometry files, have been deposited in the MetaboLights-compatible Metabolonote repository under accession number MPST000150. The data are publicly available at the Metabolonote database (https://repository.massbank.jp/). All other data supporting the findings of this study are available within the article and its Supplementary Information files.
