# Correction: Hypoxia and aspirin additively increase intracellular glutamine accumulation in *PIK3CA*-mutated colorectal cancer cells

**DOI:** 10.1038/s41598-026-65164-6

**Published:** 2026-08-31

**Authors:** Nodoka Umezaki, Shogen Boku, Yoshiyuki Matsuo, Tetsushi Yamamoto, Hironaga Satake, Motoki Watanabe, Kiichi Hirota, Tomoharu Sugie, Mitsugu Sekimoto

**Affiliations:** 1 Department of Surgery, Kansai Medical University, Hirakata City, Osaka 573-1191 Japan; 2 Cancer Treatment Center, Kansai Medical University Hospital, 2-3-1 Shinmachi, Hirakata City, Osaka 573-1191 Japan; 3 Department of Human Stress Response Science, Institute of Biomedical Science, Kansai Medical University, Hirakata City, Osaka 573-1191 Japan; 4 Pathological and Biomolecule Analyses Laboratory, Faculty of Pharmacy, Kindai University, Higasiosaka City, Osaka 577-8502 Japan; 5 Department of Medical Oncology, Kochi Medical School, Kochi University, Nankoku City, Kochi 783-8505 Japan; 6 Department of Molecular-Targeting Prevention, Kyoto Prefectural University of Medicine, Kamigyo-ku, Kyoto 602-8566 Japan

Correction to: *Scientific Reports* https://doi.org/10.1038/s41598-026-42753-z, published online 24 March 2026

This Article contained an error in Figure 5. During figure assembly, the same bar graph was inadvertently used for panels 5a, 5b, and 5c, causing the panels to appear identical despite representing different experimental conditions.

The original Fig. 5 and accompanying legend appears below.

**Figure 5:**
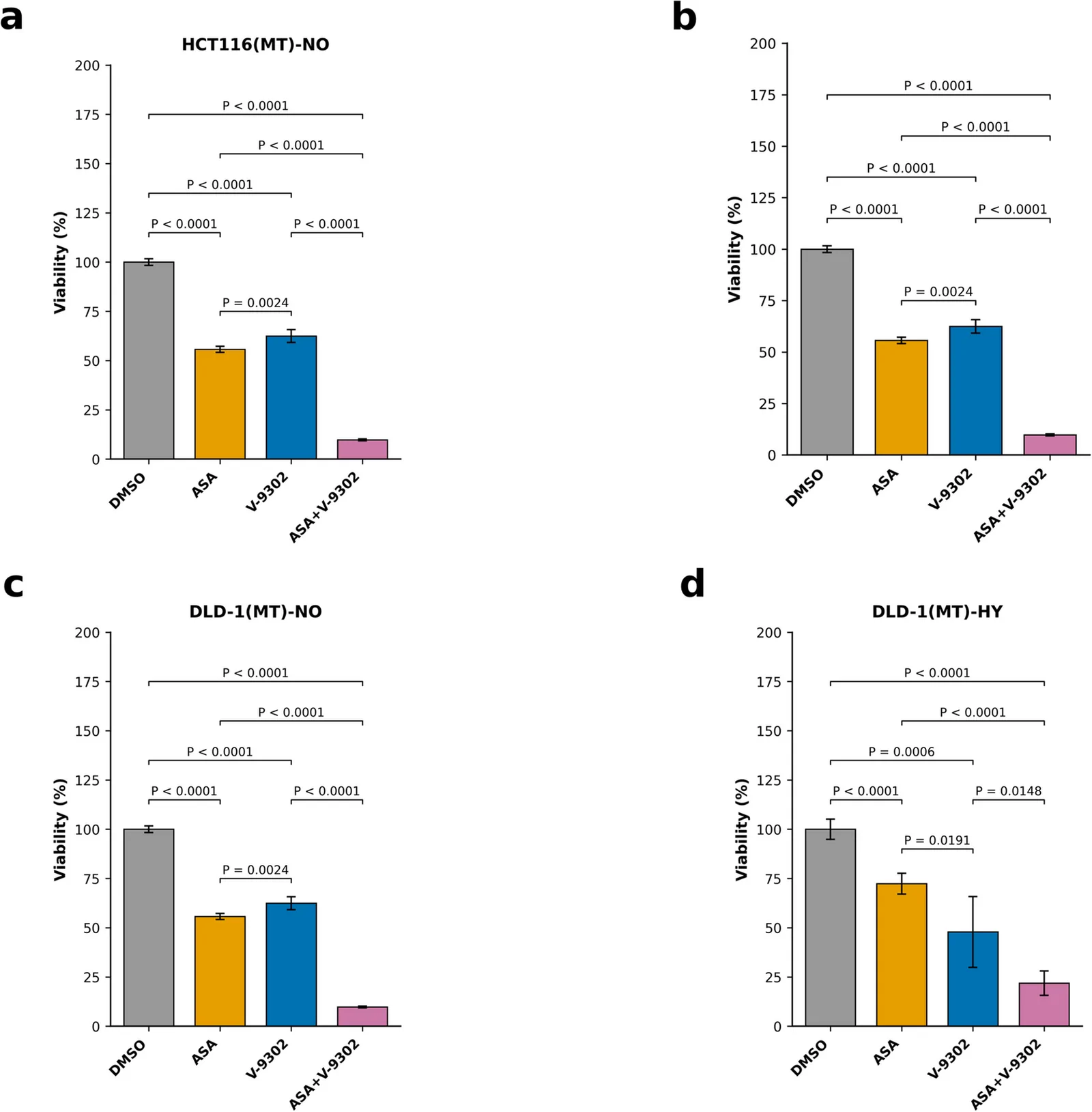
Combinatorial effects of aspirin and V-9302 on the viability of *PIK3CA*-mutant colorectal cancer cells. Cell viability was assessed using the CCK-8 assay after 72 h of incubation with 2 mM aspirin (ASA) and/or 20 µM V-9302. (**A**,** B**) *PIK3CA*-mutant (MT) HCT116 cells and (C, D) *PIK3CA*-MT DLD-1 cells were evaluated under (**A**,** C**) normoxic (20% O_2_) and (**B**,** D**) hypoxic (1% O_2_) conditions. Each independent biological experiment was performed in technical duplicates to ensure intra-experimental consistency. Results are expressed as the mean ± S.D. (*n* = 3 independent biological replicates). Statistical significance was determined using one-way ANOVA followed by Tukey’s post-hoc test.

The original Article has been corrected.

