# Supplementary figures and images for: Hypoxia and aspirin additively increase intracellular glutamine accumulation in PIK3CA-mutated colorectal cancer cells
Source: Sci Rep. 2026 Mar 24;16:9202. doi: 10.1038/s41598-026-42753-z (PMC13013922; doi:10.1038/s41598-026-42753-z)

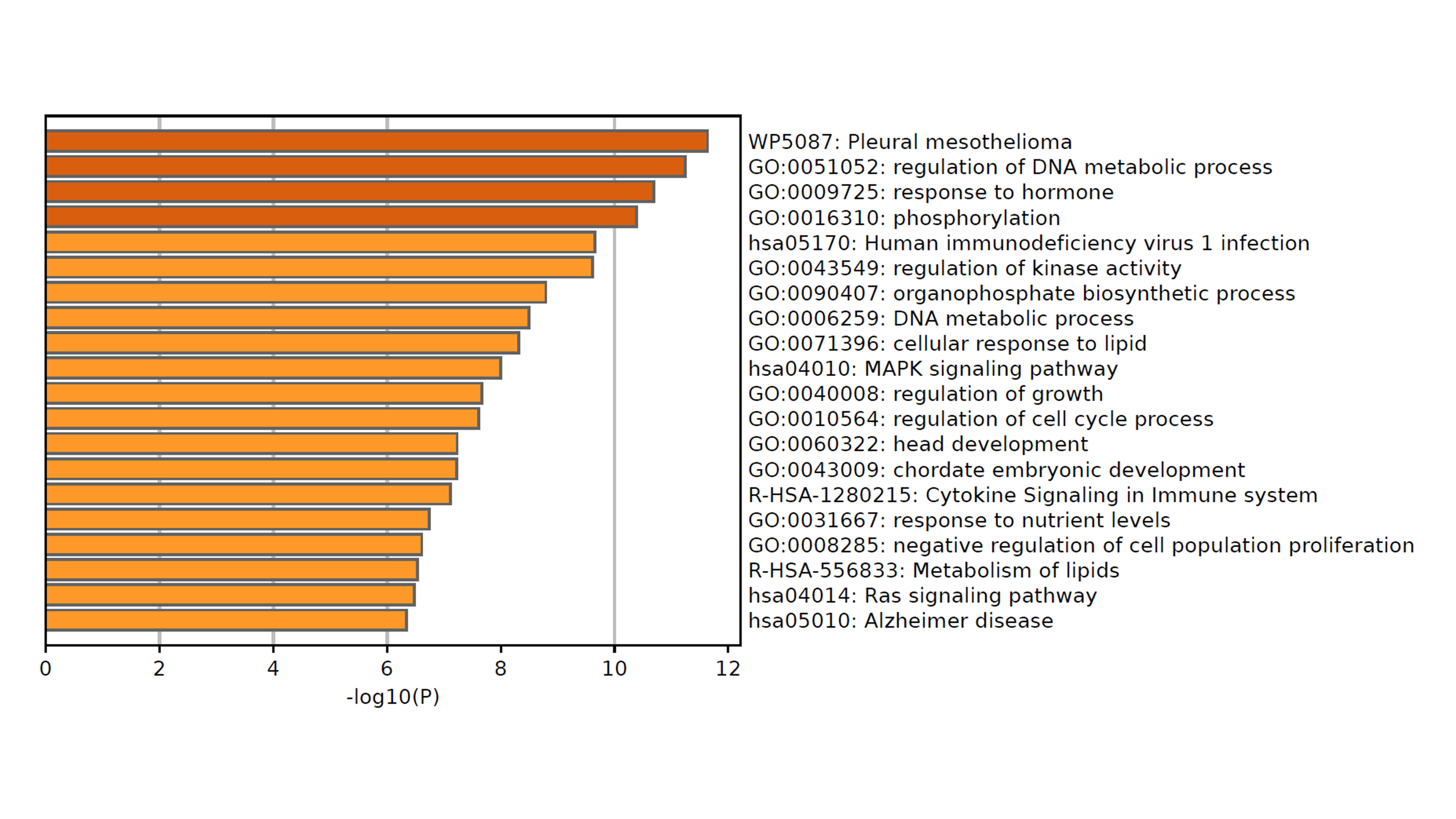

Supplement: Supplementary file 1 — Supplementary Material 1 [file 41598_2026_42753_MOESM1_ESM.tif]

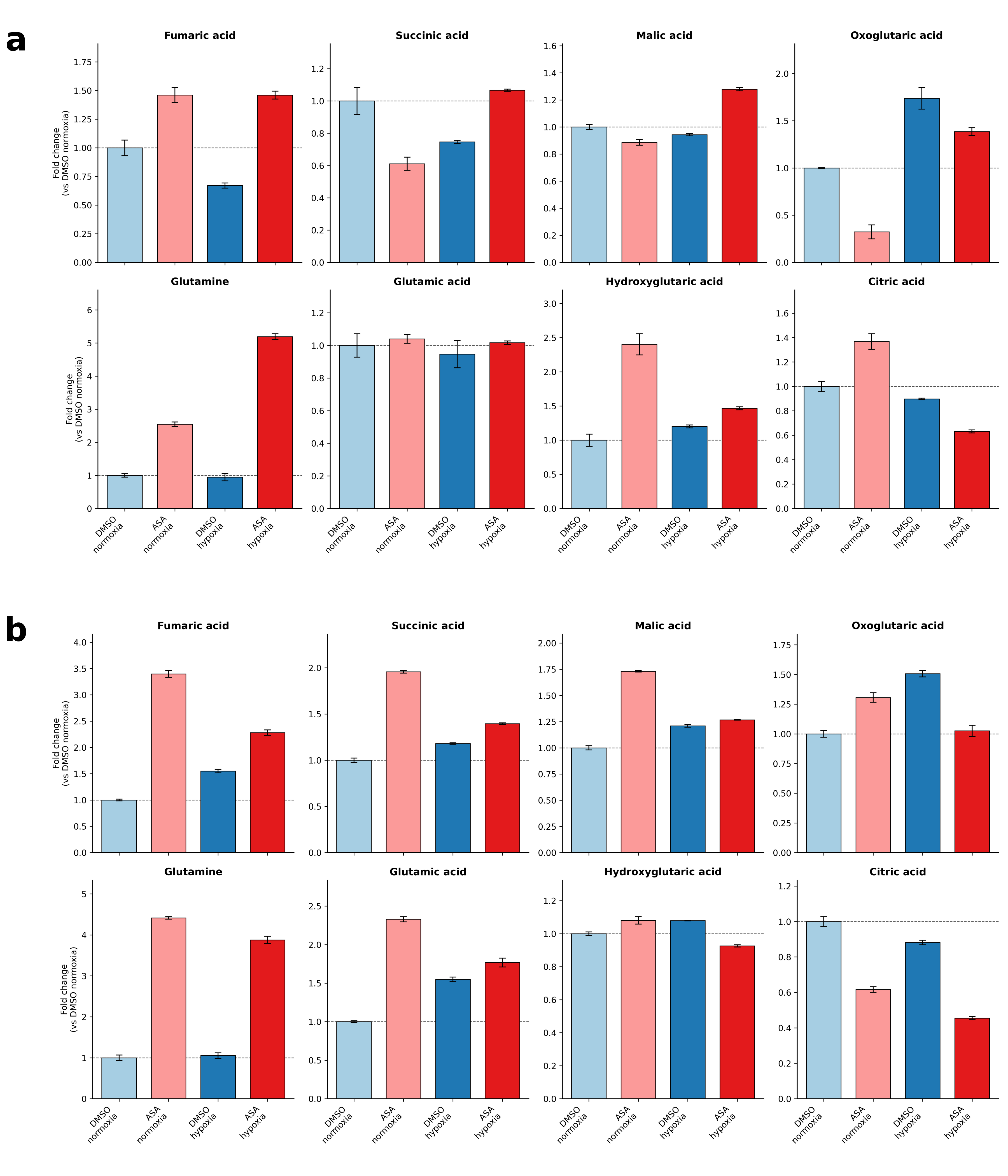

Supplement: Supplementary file 2 — Supplementary Material 2 [file 41598_2026_42753_MOESM2_ESM.tiff]

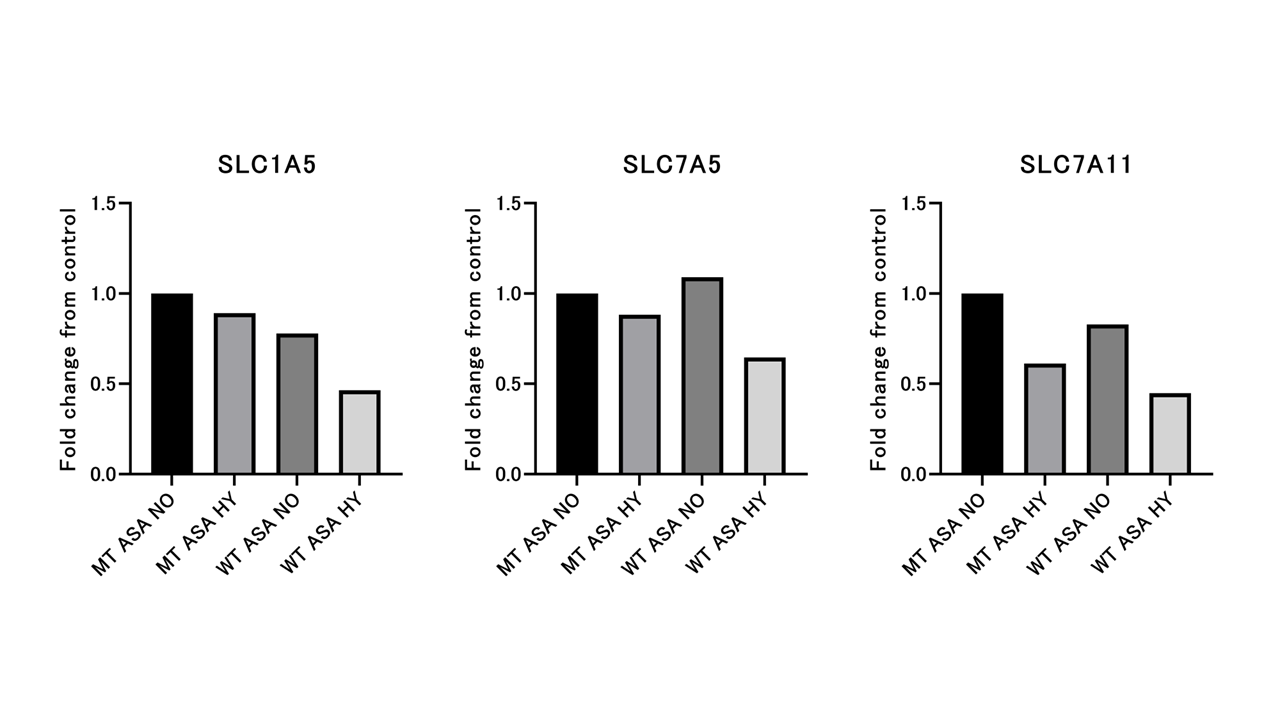

Supplement: Supplementary file 3 — Supplementary Material 3 [file 41598_2026_42753_MOESM3_ESM.tif]

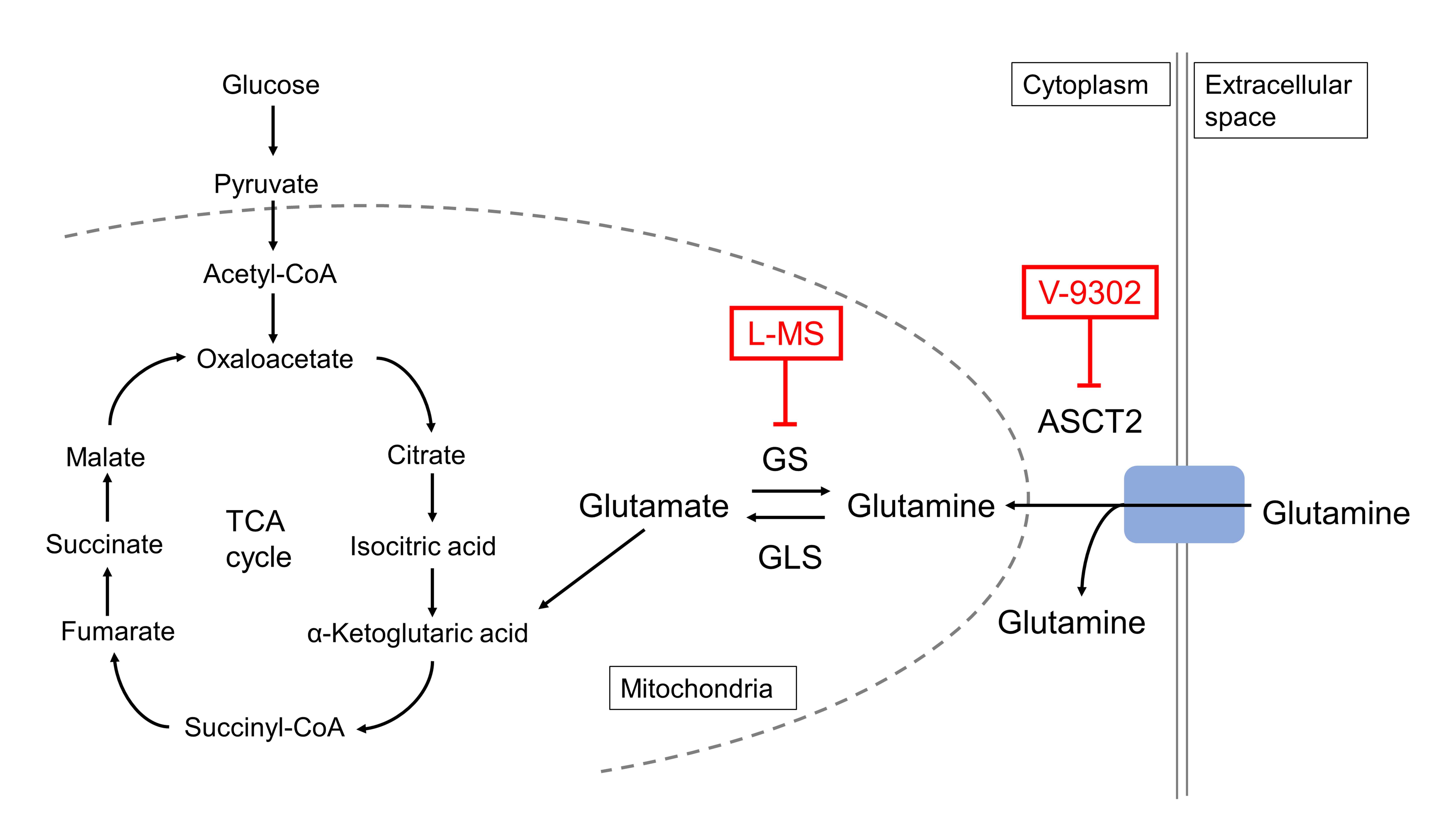

Supplement: Supplementary file 4 — Supplementary Material 4 [file 41598_2026_42753_MOESM4_ESM.tif]

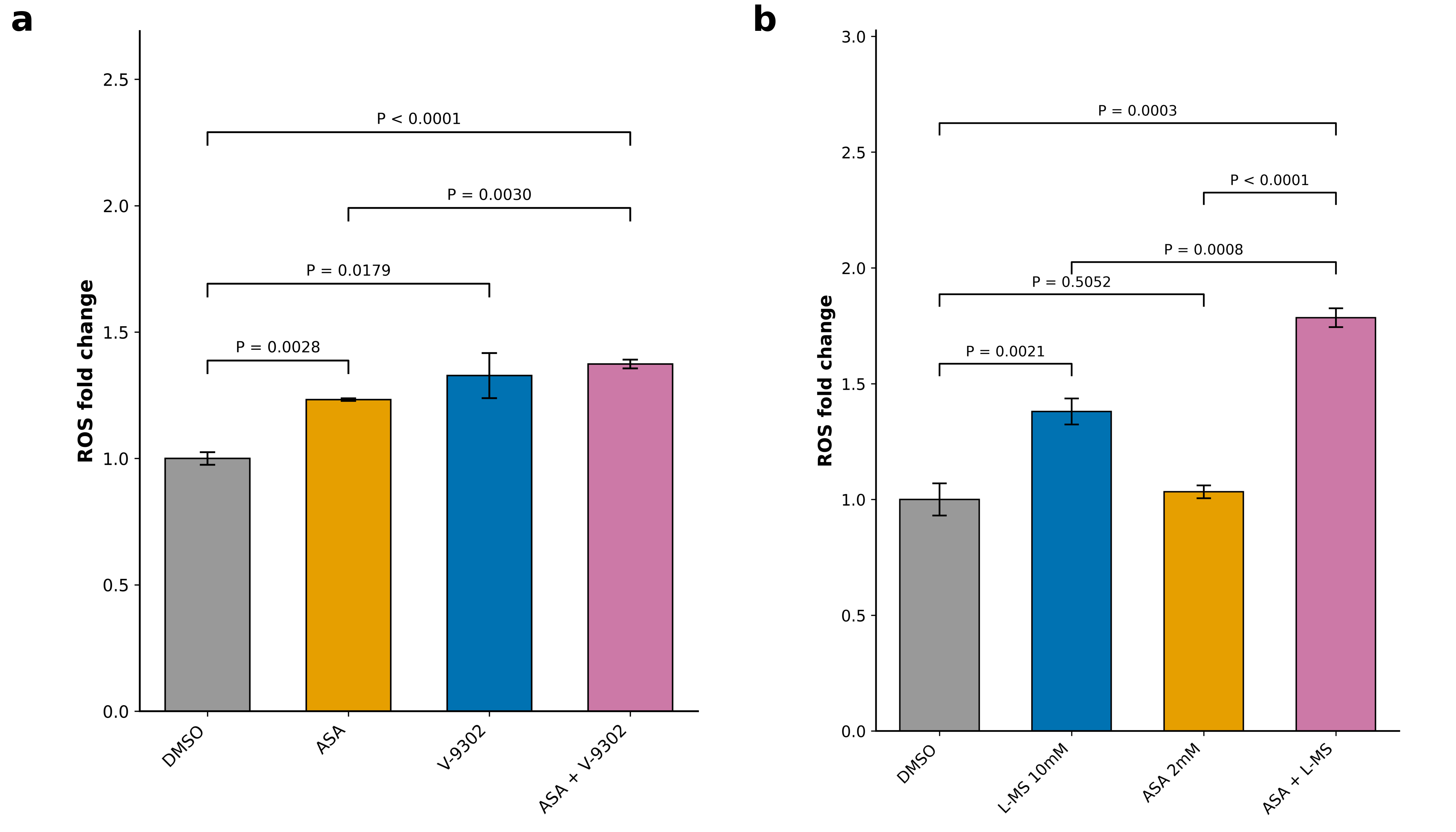

Supplement: Supplementary file 5 — Supplementary Material 5 [file 41598_2026_42753_MOESM5_ESM.tiff]

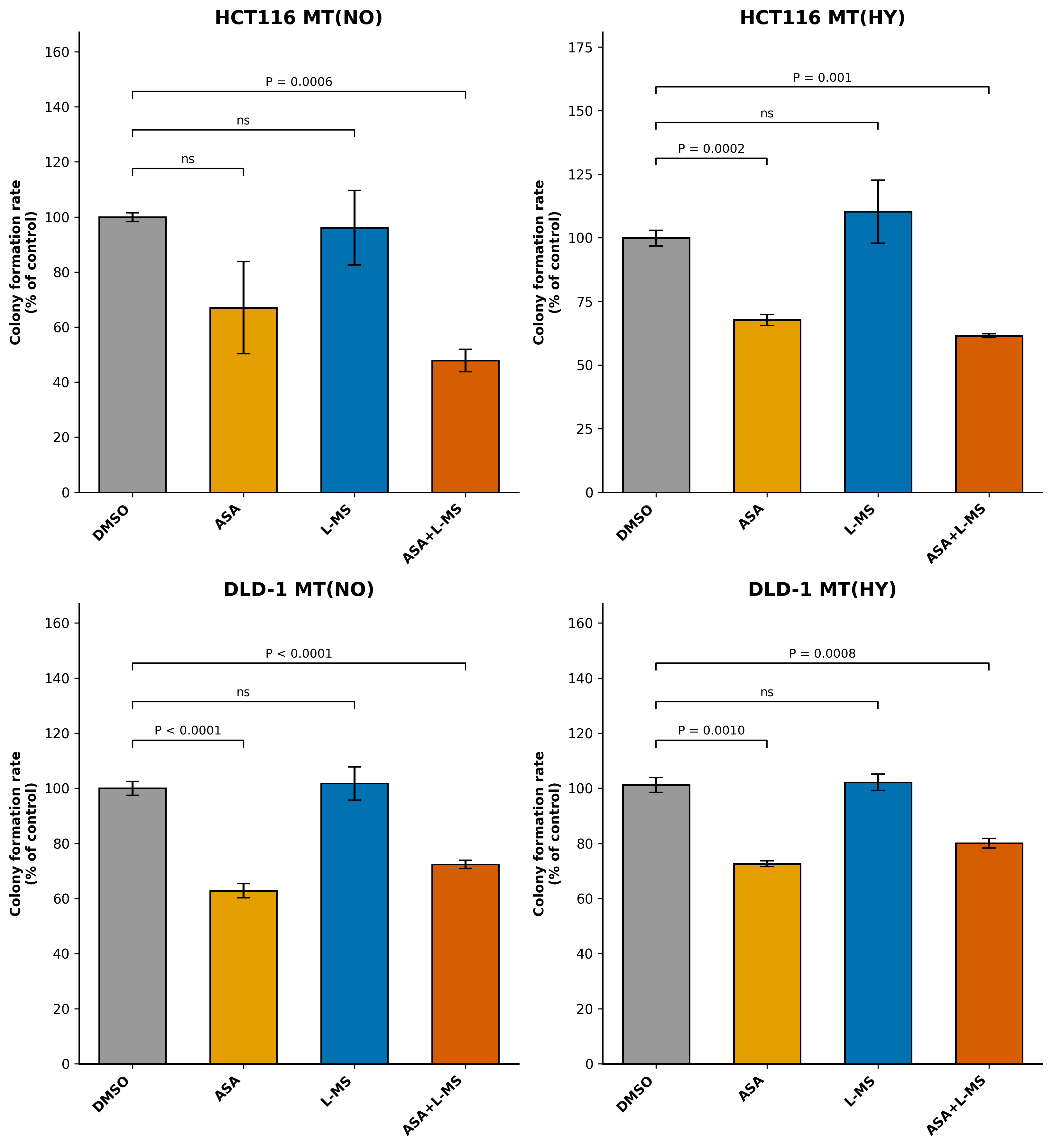

Supplement: Supplementary file 6 — Supplementary Material 6 [file 41598_2026_42753_MOESM6_ESM.tiff]
