# Supplementary material for: Hypoxia and aspirin additively increase intracellular glutamine accumulation in PIK3CA-mutated colorectal cancer cells
Source: Sci Rep. 2026 Mar 24;16:9202. doi: 10.1038/s41598-026-42753-z (PMC13013922; doi:10.1038/s41598-026-42753-z)
Supplement: Supplementary file 7 — Supplementary Material 7 [file 41598_2026_42753_MOESM7_ESM.docx]

| **Analytes** | **Precursor ion**  **(m/z)** | **Product ion**  **(m/z)** | **Collision energy**  **(eV)** | **Detection mode** |
| --- | --- | --- | --- | --- |
| **Fumaric Acid** | **115.16** | **71.11** | **10.25** | **Negative** |
| **Succinic Acid** | **117.21** | **73.18** | **10.25** | **Negative** |
| **Oxaloacetic Acid** | **131.04** | **87.11** | **10.25** | **Negative** |
| **Malic Acid** | **133.11** | **115.00** | **10.25** | **Negative** |
| **2-Oxoglutaric Acid** | **145.02** | **101.00** | **10.25** | **Negative** |
| **L-Glutamine** | **147.10** | **129.90** | **10.25** | **Positive** |
| **L-Glutamic Acid** | **148.10** | **84.11** | **14.60** | **Positive** |
| **L-α-Hydroxyglutaric acid** | **147.09** | **129.00** | **10.25** | **Negative** |
| **Trans-Aconitic Acid** | **172.95** | **129.00** | **10.25** | **Negative** |
| **Citric Acid** | **190.97** | **85.11** | **15.66** | **Negative** |
| **Isocitric acid** | **190.97** | **85.11** | **15.66** | **Negative** |
