# Supplementary material for: Hypoxia and aspirin additively increase intracellular glutamine accumulation in PIK3CA-mutated colorectal cancer cells
Source: Sci Rep. 2026 Mar 24;16:9202. doi: 10.1038/s41598-026-42753-z (PMC13013922; doi:10.1038/s41598-026-42753-z)
Supplement: Supplementary file 10 — Supplementary Material 10 [file 41598_2026_42753_MOESM10_ESM.docx]

**Supplementary Information Captions**

**Fig. S1 Enrichment analysis of “aspirin-related genes” in MCF-7 cells based on Connectivity Map data.**

**Fig. S2 A quantitative assay for TCA cycle metabolites was used to measure TCA cycle metabolites using LC-MS/MS.** The data obtained with DMSO-treated controls under normoxia were considered to have a value of 1. (A) *PIK3CA*-MT HCT116 and (B) *PIK3CA*-WT HCT116colorectal cancer cells were used to measure TCA cycle metabolites using LC-MS/MS. **Data represent the mean ± S.D. (*n = 3* independent biological replicates).**

**Fig. S3 Amino acid-related gene expression of in HCT116 *PIK3CA* MT/WT cell lines in qRT-PCR.**

**Fig. S4 Schematic illustration of the sequential enzymatic reactions of glutamine metabolism with inhibitors for each step.**

**Fig. S5 ROS levels were increased following exposure to aspirin in *PIK3CA*-MT HCT116 cells.** Co-treatment with aspirin and either (A) V-9302 (20 µM) or (B) L-MS (10 mM) resulted in a more pronounced increase in intracellular ROS levels. **Data reflect raw relative luminescence units (RLU) representing the total cumulative oxidative burden within the niche. Data represent the mean ± S.D. from *n = 3* independent biological replicates.**

Statistical analyses were performed using one-way ANOVA with Tukey’s post-hoc test.

**Fig. S6 Impact of aspirin and L-MS co-treatment on the cell viability of *PIK3CA*-MT cells.** *PIK3CA*-MT HCT116 and DLD-1 cells were treated with 2 mM aspirin (ASA) and/or L-MS (10 mM) under normoxic (20% O^2^) and hypoxic (1% O^2^) conditions for 72 h. **Each independent biological experiment was performed in technical duplicates. Data represent the mean $\pm$ S.D. from *n = 3* independent biological replicates.** Statistical analyses were performed using one-way ANOVA with Tukey’s post-hoc test.

**Table S1 Targeted Metabolomics Parameters** This table provides a summary of the MS parameters used for multiple reaction monitoring (MRM) transitions (precursor and product ions) and collision energies for each TCA cycle metabolite.

**Table S2 List of aspirin-related genes.** List of genes in HT-29 (A), in the eight cell lines (B), and MCF7 (C)

**Table S3 Detailed results of enrichment analysis in HT-29 (A) and in the eight cell lines (B).**
